# Physical Performance as a Predictor of Length of Hospital Stay in Patients Undergoing Open-Heart Surgery: A Multicenter Prospective Study

**DOI:** 10.3390/medsci14020334

**Published:** 2026-06-20

**Authors:** Wararat Tavonudomgit, Kornanong Yuenyongchaiwat, Lucksanaporn Mahawong, Khanistha Wattanananont, Chitima Kulchanarat, Sasipa Buranapuntalug, Opas Satdhabudha

**Affiliations:** 1 Physiotherapy Department, Faculty of Allied Health Sciences, Thammasat University, Pathum Thani 12120, Thailand; 2 Thammasat University Research Unit in Physical Therapy in Respiratory and Cardiovascular Systems, Thammasat University, Pathum Thani 12120, Thailand; 3 Cardiac Rehabilitation Unit, Excellence Center, Faculty of Medicine, Navamindradhiraj University, Bangkok 10300, Thailand; 4 Physical Therapy Center, Thammasat University Hospital, Pathum Thani 12120, Thailand; 5 Department of Surgery, Faculty of Medicine, Thammasat University, Pathum Thani 12120, Thailand

**Keywords:** physical performance, cardiac surgery, cardiovascular disease, prediction, morbidity, length of hospital stay

## Abstract

Background: Patients undergoing open-heart surgery (OHS) are at risk of postoperative morbidity and mortality. Physical performance has been increasingly recognized as an important factor influencing postoperative outcomes. Therefore, the study aimed to investigate the associations and predictive value of physical performance on postoperative complications and duration of hospital stay. Methods: A prospective cohort study was conducted in 116 patients who were admitted to OHS. Preoperative assessment of physical performance, i.e., Short Physical Performance Battery (SPPB), Five Times Sit to Stand Test (5STS), gait speed (5 m walk test: 5MWT), Timed Up and Go (TUG), and handgrip strength. Duration of hospital stay and incidence of post-operative complications were recorded. Differences between participants with and without postoperative complications were analyzed using independent samples t-tests for continuous variables and chi-square tests for categorical variables. The associations between physical performance and postoperative outcomes were assessed using Spearman’s rank correlation coefficient. Hierarchical regression analysis was conducted to determine the predictive contribution of physical performance. Results: A total of 116 participants were submitted for OHS in two medical school hospitals; however, 108 individuals completed the pre-operative physical performance. The most common procedures were coronary artery bypass grafting and valve surgery. Fifty-one participants (47.22%) experienced postoperative complications, including five deaths, corresponding to 4.63% mortality. For the length of hospital stay analysis, five participants who died postoperatively were excluded, resulting in a final sample of 103 participants. Physical performance was significantly associated with the length of hospital stay (*p* < 0.05). Hierarchical regression analysis showed that the final prediction model explained 13.4% of the variance in length of hospital stay, with SPPB independently contributing an additional 6.0% to the model, followed by 5STS, 5MWT, handgrip strength, and TUG, which accounted for an additional 5.1%, 4.6%, 4.4%, and 3.7%, respectively. Conclusions: Preoperative physical performance was associated with length of hospital stay. While each measure explained a relatively small proportion of the variance in hospital stay, these assessments offer a simple, non-invasive, and clinically feasible approach to evaluating functional reserve before surgery. These findings highlight the importance of incorporating functional assessment into perioperative care to support risk stratification and guide rehabilitation strategies.

## 1. Introduction

Globally, cardiovascular diseases (CVDs) are a major cause of mortality. From 2010 to 2020, there was an approximate 18.71% increase in the number of deaths from CVDs [1]. Open-heart surgery, including coronary artery bypass graft surgery (CABG) and heart valve surgery (e.g., valve repair, valve replacement), is a common and essential treatment for advanced CVD. However, patients undergoing open-heart surgery remain at high risk of postoperative complications due to prolonged invasive mechanical ventilation, length of hospital stay, or increased mortality rate [2,3,4].

Physical performance has emerged as an important indicator of functional capacity and physiological reserve in patients with cardiovascular disease. O’Neill DE et al. [5] reported that patients with heart disease experienced a 25–50% decrease in their physical performance. The Short Physical Performance Battery (SPPB) was a commonly used assessment tool for physical performance and widely applied in clinical settings to estimate the prolonged length of hospital stay in older patients undergoing CABG [6]. In addition, previous studies have shown that patients with CVD often exhibit reduced muscle strength and impaired functional performance, which may contribute to poorer postoperative outcomes [6,7,8,9,10].

Given the growing recognition of physical performance as a potential predictor of clinical outcomes, preoperative risk assessment is essential for patients undergoing open-heart surgery. Early identification of individuals with reduced functional capacity may facilitate targeted interventions, such as prehabilitation and early mobilization, to improve recovery and reduce complications. Thus, physical performance assessment before open-heart surgery may be a benefit in patients after surgery, and these can lead to early intervention, including awareness and prevention of problems. However, in Thailand, routine preoperative functional assessment and perioperative rehabilitation are not consistently implemented across clinical settings, and evidence on the predictive value of physical performance measures in patients undergoing open-heart surgery remains limited. In addition, differences in healthcare systems, perioperative management, rehabilitation practices, and patient characteristics may influence postoperative recovery and length of hospital stay. Therefore, the study aimed to (1) determine the correlation between physical performance and postoperative outcomes, including length of hospital stay, and (2) identify factors that predict length of hospital stay in patients undergoing open heart surgery.

## 2. Materials and Methods

A prospective cohort study was conducted to examine the predictive value of physical performance for length of hospital stay among patients admitted for open-heart surgery in two medical school hospital settings. Participants were recruited from Thammasat University Hospital and Vajira Hospital because both are tertiary care teaching hospitals with specialized cardiovascular and cardiothoracic surgery services, providing a large number of patients undergoing open-heart surgery. In addition, the inclusion of two centers helped improve the generalizability of the findings across different clinical settings and perioperative care practices.

The sample size was calculated using G*Power version 3.1.9.4. Based on a previous study by Cordeiro et al. [11], which reported a correlation coefficient of r = 0.27 between gait speed and length of hospital stay in patients awaiting open-heart surgery, a minimum sample size of 105 participants was required, assuming a significance level (α) of 0.05 and a statistical power of 0.80. To account for potential missing data, the sample size was increased by 10%, resulting in a total required sample of 116 participants.

The study was conducted in accordance with the Declaration of Helsinki, the Belmont Report, CIOMS Guidelines, and the International Practice (ICH-GCP). In addition, this study was approved by the Human Research Ethics Committee of Thammasat University (Science), approval reference COA No. 075/2567, and by the Institutional Review Board of the Faculty of Medicine Vajira Hospital, approval reference COA No. 069/2568. All participants received the information sheet and were required to complete an informed consent form before participation.

Patients who underwent open-heart surgery aged 20 years or older, both male and female, were enrolled. However, patients who had unstable angina or arrhythmia 24 h before the test, resting heart rate over 120 bpm, had fever (defined as 37.5 degree Celsius), systolic blood pressure over 180 mmHg or/and diastolic blood pressure over 100 mmHg 24 h before the test, had other surgeries (e.g., thoracotomies), history of stroke or previous heart surgery, or unable to walk were excluded. In addition, participants who died after surgery were excluded from the study. One or two days before the operation, all participants were required to perform the physical performance. Duration of hospitalization and morbidity complications during hospitalization were recorded.

All physical performance assessments were performed using standardized testing protocols. Assessors received training regarding test procedures and data collection prior to study implementation, ensuring consistency across the two study centers. The SPPB, Timed Up and Go (TUG), Five Times Sit to Stand Test (5STS), gait speed (5 m walk test: 5MWT), and handgrip strength were selected in the present study due to their strong clinical relevance, feasibility, and established validity in assessing functional capacity in patients with cardiovascular disease. Further, these measures play an important key component of physical performance, including mobility, balance, lower extremity strength, and overall functional status, which are essential for postoperative recovery [6,8,12,13,14,15,16,17]. TUG reflects functional mobility and dynamic balance [18,19], while 5STS primarily assesses lower limb muscle strength and functional endurance [17]. SPPB provides a comprehensive evaluation by integrating balance, gait speed, and strength [6]. Further, gait speed alone is widely recognized as a simple indicator of overall health and functional reserve [14]. Handgrip strength is also a representation of muscle function and overall physical capacities [16]. Importantly, these tests are quick, non-invasive, and require minimal equipment, making them highly feasible in clinical settings, particularly in preoperative assessment. In addition, these assessments have also been widely used and validated in previous studies as predictors of adverse outcomes, including prolonged length of hospital stay, postoperative complications, and mortality in surgical and cardiac populations [6,8,12,13,15,16,17].

The SPPB consists of three performance tests: gait speed, standing balance, and repeated chair stands. The sum score ranges from 0 to 12, with each test scoring between 0 (worse) and 4 (best). A higher score indicates a higher level of function. A cutoff of SPPB score of 9.5 is considered to indicate a prolonged hospitalization [6].

The 5STS measures lower-body strength and endurance. In the starting position, participants sit in the chair with their back straight and feet flat on the floor. Their arms should be placed across their chest, with hands resting on their shoulders. Participants were asked to stand up fully and then sit down fully, repeated five times as quickly as possible [20,21].

Gait speed was measured at the time of walking in a short distance (usually 5 m) at a comfortable pace, and is one of the most commonly used tests to screen for frailty and identify high-risk older adults. The 5MWT has been recommended for use by the Society of Thoracic Surgeons [22]. Participants were instructed to walk at a comfortable pace over a distance of 5 m, and the time taken to complete the walk was recorded. The test was performed three times, and the average gait speed was calculated for analysis.

The TUG is a simple, quick, and widely used measure of lower extremity function, mobility, and risk of falls. Participants sit upright in the chair with their back supported and arms resting on the armrests. After that, individuals were asked to stand up from the chair—without using their hands if possible—walk 3 m at a comfortable pace to a marked point, turn around, walk back, and sit down in the chair [8].

Handgrip strength is measured using a Smedley handgrip dynamometer (T.K.K.5401 Grip-D; Tokyo, Japan) in this study. According to the US National Health and Nutrition Examination Survey (NHANES) protocol for using the Smedley handgrip dynamometer, the participants were required to stand with their elbow extended, hold the handgrip dynamometer with their dominant hand, and squeeze it as hard as possible for 10–15 s [23,24]. The test was repeated two times, and the highest score was recorded.

Post-operative morbidity, including post-operative atrial fibrillation, wound infection, Extracorporeal Membrane Oxygenation (ECMO), Postoperative fever defined as a body temperature ≥ 38 °C sustained for two consecutive days or a single measurement of ≥39 °C following surgery [25], medical diagnosis of delirium, hemodialysis/renal injury, reintubation, readmission to intensive care unit (ICU), and reoperation were recorded. In addition, participants who died postoperatively were included in the postoperative complication analysis; however, they were excluded from the length of hospital stay analysis because hospital discharge outcomes could not be assessed. Therefore, post-operative morbidity was also defined as a post-operative complication. Additionally, details such as the type of surgery, duration of operation, duration of mechanical ventilation, and length of hospital stay were reported.

The Kolmogorov–Smirnov statistic is used to determine the distribution of the data. Spearman’s rank correlation is used to assess the correlation between physical performance and length of hospital stay in patients admitted for open-heart surgery. Finally, a series of hierarchical regression analyses was performed to determine whether physical performance predicted length of hospital stay. Regression analyses were conducted to determine the unique contribution of physical performance to the prediction of length of hospital stay after adjustment for age and sex. The hierarchical linear regression analysis of hospitalization assessed the predictive power of sex and age (step 1), and the physical performance (step 2). Consequently, baseline sex and age were entered in step one, and the physical performance (i.e., SPPB, 5STS, gait speed, handgrip strength, or TUG) was entered in step two. Therefore, models 1–5 represent separate regression models. Because the length of hospital stay demonstrated a right-skewed distribution, a sensitivity analysis was performed using the natural logarithm-transformed length of hospital stay as the dependent variable. Hierarchical linear regression analyses were repeated using the transformed outcome to assess the robustness of the findings. Multicollinearity between physical performance and duration of hospital stay was assessed using the variance inflation factor (VIF). All data were analyzed using SPSS Version 26.0, with statistical significance set at *p*-value < 0.05.

## 3. Results

**Figure 1:**
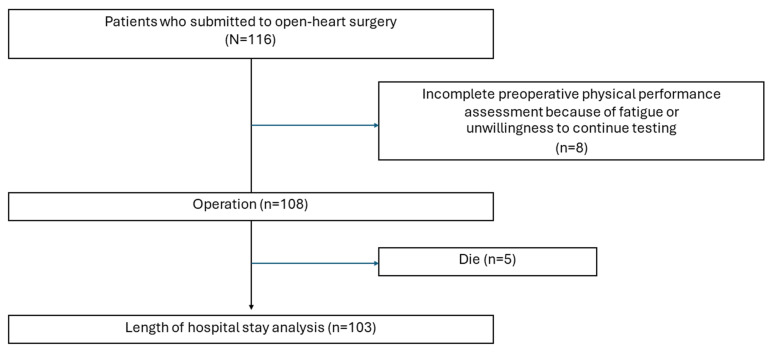
Flow chart of recruitment procedure and study profile of participants.

A total of 116 participants were included in the analysis. However, eight participants did not complete all five physical performance tests because of fatigue (n = 3) and unwillingness to continue testing (n = 5), resulting in incomplete data for the planned analyses. Therefore, 108 participants were analyzed. In addition, participants who died postoperatively were included in the postoperative complication analysis; however, they were excluded from the length of hospital stay analysis because hospital discharge outcomes could not be assessed (Figure 1).

Nearly half of the participants (47.22%) experienced postoperative complications, while five patients (4.63%) died. Consequently, 52.8% of the participants had no postoperative complications. The most common complication was postoperative atrial fibrillation, observed in 31 participants (28.7%). Postoperative fever and the need for hemodialysis were each reported in 13 participants (12.0%). Reintubation occurred in 6 participants (5.6%), while 5 participants (4.6%) required ICU readmission. Reoperation was necessary in 3 cases (2.8%), and delirium was observed in 2 participants (1.9%). One participant (0.9%) required ECMO. No cases of sternal wound infection were reported. The results demonstrated that patients in the complication group exhibited significantly older age and lower physical performance compared to those without complications.

There was a significant association between the type of surgery and postoperative complications. Patients who underwent combined procedures had a higher proportion of postoperative complications compared with those without complications, whereas CABG was more common among patients without complications (Table 1).

Due to five participants dying, the length of hospital stay was analyzed in 103 participants. The length of hospital stay was significantly correlated with multiple physical performance measures, including SPPB, TUG, 5STS, 5MWT, and handgrip strength, with generally weak correlations. Furthermore, older age, lower SPPB scores, longer 5STS duration, prolonged TUG time, slower gait speed, and reduced handgrip strength were associated with an increased risk of postoperative complications (Table 2).

To investigate whether physical performance predicts duration of hospital stay, a series of hierarchical regression analyses was performed (Table 3). Age and sex (step one) predicted length of hospital stay, accounting for 7.4% of the variance. At step two, SPPB accounted for an additional 6.0% of the variance; together, all predictors accounted for 13.4% of the variance in duration of hospital stay, followed by 5STS (5.1%), 5MWT (4.6%), handgrip strength (4.4%), and TUG (3.7%). Further, multicollinearity diagnostics indicated no evidence of multicollinearity among the independent variables, as the variance inflation factor (VIF) was low (VIF = 1.01–1.53), suggesting acceptable independence between predictors. In addition, sensitivity analyses using log-transformed length of hospital stay yielded results similar to those obtained from the original models. The direction and statistical significance of the associations between physical performance measures and length of hospital stay remained unchanged, indicating that the findings were robust to the distribution of the outcome parameter.

## 4. Discussion

The present study explored the correlation between physical performance before open-heart surgery and postoperative complications and length of hospital stay. The present study demonstrated significant, albeit weak, correlations between physical performance measures and clinical outcomes after postoperative open-heart surgery, including length of hospital stay and postoperative complications. Physical performance was significantly associated with hospital stay, suggesting that reduced physical performance is related to prolonged hospitalization. In addition, those physical performances could predict hospital stay among patients who underwent open-heart surgery. However, the strength of these correlations was generally small, indicating that physical performance represents only one component within a multifactorial clinical context.

The incidence of post-operative morbidity and mortality in patients undergoing open-heart surgery is high. These findings are consistent with previous studies indicating that patients undergoing open-heart surgery are at high risk and are associated with substantial postoperative morbidity and mortality. Common complications include approximately 32% for atrial fibrillation, 8.9% for prolonged mechanical ventilation, and 3.3% for both renal failure and reoperation due to bleeding [26]. Additional complications, such as prolonged ECMO support, fever, postoperative delirium, reintubation, ICU readmission, and reoperation, have also been documented to affect recovery and prolong hospitalization adversely [27].

Regarding preoperative physical performance and postoperative complications, the present study demonstrated that associations were observed between physical performance measures and postoperative outcomes, albeit relatively weak. Several factors may explain these findings. The length of hospital stay following open-heart surgery is a multifactorial outcome influenced not only by physical performance but also by surgical complexity, perioperative complications, comorbidities, postoperative management, discharge planning, and institutional protocols [28,29]. These factors may reduce the magnitude of the relationship between preoperative functional status and hospital outcomes. Further, studies conducted in Western countries included older populations with higher frailty prevalence, greater functional limitations, reduced physiological reserve, and higher rates of comorbidities [30]. In contrast, the participants in the present study were relatively younger, which may have reduced the overall severity of physical impairment. Additionally, institutional factors, including postoperative rehabilitation practices, ICU management, and discharge criteria across the participating hospitals, may have affected hospital stay independently of physical performance status. Therefore, while physical performance measures demonstrated statistically significant associations with postoperative outcomes, their predictive contribution should be interpreted within the broader context of perioperative and healthcare system factors.

SPPB, which integrates balance, gait speed, and lower extremity strength, was associated with operative and hospital outcomes. Lower SPPB scores indicate reduced functional status and have been linked to a higher risk of postoperative complications and mortality; Guralnik et al. [31] demonstrated that SPPB is a robust predictor of disability and adverse health outcomes, while subsequent studies have extended its predictive value to surgical populations. Further, each 1-point decrease in SPPB score was associated with a 10% increase in the risk of adverse outcomes after surgery [9]. Therefore, lower SPPB scores are associated with frailty, prolonged hospitalization, and poorer postoperative recovery among cardiac surgery patients.

The 5STS, reflecting lower limb muscle strength and functional endurance, showed significant associations and contributed to the predictive model. Poor performance in 5STS suggests reduced muscle strength and functional independence, which may delay postoperative recovery and mobilization. A systematic review and meta-analysis by Muñoz-Bermejo et al. [21] reported that 5STS is a reliable indicator of lower extremity strength and functional limitation. In addition, the 5STS serves as an efficient tool for assessing functional capacity in incentive care unit patients, for which prolonged 5STS performance has been significantly associated with reduced lower limb muscle strength and also longer hospital stays [17]. Further, the 5STS has been used for sarcopenia assessments as an indicator of poor physical performance. Impaired 5STS performance reflects reduced muscle strength and functional decline [32,33], which may contribute to prolonged hospitalization in patients following cardiac surgery.

Gait speed, often considered a “vital sign” of functional status, was also significantly associated with prolonged hospitalization. Slow gait speed reflects impairments in multiple systems, including lower limb muscle strength, cardiopulmonary function, and neuromuscular coordination, all of which are essential for recovery after major surgery. Reduced gait speed indicates generalized sarcopenia or physical frailty, particularly in poor physical performance [32,33,34]. Evidence highlighted its strong predictive value for adverse survival outcomes and in-hospital mortality and morbidity in CAD patients or patients undergoing cardiac surgery [35,36]. Several large studies have demonstrated that reduced gait speed is independently associated with adverse outcomes after cardiac surgery. Systematic reviews and meta-analyses in seven studies reported that slower gait speed significantly increases the risk of in-hospital mortality, major morbidity, acute kidney injury, prolonged ventilation, and longer ICU stay [37]. In addition, patients with slow gait speed were associated with a higher risk of in-hospital mortality, with a risk ratio of 2.32 [37]. Each small reduction in gait speed (0.1 m/s) further increases mortality risk, highlighting its sensitivity as a prognostic indicator of operative mortality in cardiac surgery [38]. Therefore, gait speed is not only a simple functional measure but also a predictor of complications and prolonged hospitalization after cardiac surgery, as it reflects the patient’s ability to recover from surgical stress.

TUG, a widely used measure of functional mobility and balance, was identified as both a correlate and a predictor of postoperative complications [39,40,41]. Prolonged TUG time reflects impaired mobility and reduced neuromuscular coordination, which may predispose patients to complications such as delayed ambulation, pulmonary dysfunction, and increased dependency. Previous studies have reported that TUG is a strong predictor of postoperative morbidity and prolonged hospital stay, particularly in older adults and cardiac surgery populations [8,18].

Handgrip strength was significantly associated with length of hospital stay in patients undergoing open-heart surgery, with lower values observed in those who developed postoperative complications. This finding is consistent with previous studies demonstrating that greater preoperative grip strength is associated with shorter hospitalization and a higher likelihood of discharge in older patients [42,43]. Likewise, reduced handgrip strength at hospital admission has been linked to prolonged hospital stay, increased postoperative morbidity, and higher mortality risk [44,45]. Poor handgrip strength is strongly associated with frailty, sarcopenia, and adverse clinical conditions, including poorer functional recovery [46,47,48,49]. Overall, preoperative assessment of handgrip strength provides valuable prognostic information and may help identify high-risk patients who could benefit from targeted rehabilitation and perioperative interventions to enhance recovery and reduce hospital stay.

The ability of physical performance, such as SPPB, gait speed, TUG, 5STS, and handgrip strength, to predict length of hospital stay in patients undergoing open-heart surgery can be explained by overall physiological reserve, functional status, and adverse outcomes [50], which is associated with an increased risk of postoperative complications and recovery after major surgery [51]. Patients with better physical performance typically have greater cardiovascular fitness and muscle strength, enabling faster recovery and fewer complications [30,52]. In addition, SPPB, gait speed, TUG, 5STS, and handgrip strength reflect key components of frailty and sarcopenia, including reduced muscle strength and impaired mobility. Frailty and sarcopenia have been widely shown to predict adverse surgical outcomes, including longer hospitalization, increased complications, and higher mortality [47,53,54]. Therefore, these measures may indirectly predict length of hospital stay by reflecting underlying physiological vulnerability and reduced functional reserve.

Several limitations should be noted that might affect the interpretation of the study. The length of hospital stay following open-heart surgery is influenced by multiple factors beyond physical performance, including intraoperative factors, e.g., surgical procedures, perioperative complications, comorbidities, detailed operative risk scores (e.g., EuroSCORE II or Society of Thoracic Surgeons Risk Score: STS risk scores or the American Society of Anesthesiologists (ASA) physical status classification system) or postoperative management, which are known to influence length of hospital stay and complications. Further, these factors may contribute to physical performance, reducing their independent predictive value in multivariate models. In the present study, data were not recorded; therefore, further study needs to be explored. In addition, participants who died postoperatively were excluded from the length of hospital stay analysis. This exclusion may have introduced survivorship bias and potentially underestimated the relationship between postoperative complications and hospital stay, as patients with the most severe clinical outcomes were not included in the analysis. Future studies with larger sample sizes should consider the use of sensitivity analyses or competing-risk models to better account for postoperative mortality when evaluating hospital length of stay. Although the sample size was calculated appropriately, it may still be insufficient to detect small effect sizes, particularly in multivariate analyses. The relatively modest sample size and variability within the study population may limit statistical power to detect independent effects, particularly for measures with smaller effect sizes. Although standardized assessment protocols and assessor training were implemented at both centers, formal inter- and intrarater reliability testing was not conducted. Therefore, potential in measurement variability between assessors and study sites cannot be warranted. Finally, other potential confounding factors, such as nutritional status, psychosocial factors, and hospital discharge policies, were not fully controlled and may have influenced the outcomes. Despite these limitations, this study provides valuable insights into the role of physical performance in predicting postoperative outcomes and highlights the importance of functional assessment in patients undergoing open-heart surgery.

## 5. Conclusions

The findings of this study indicate that physical performance measures, including SPPB, gait speed, TUG, 5STS, and handgrip strength, are significant predictors of length of hospital stay in patients undergoing open-heart surgery. Although the predictive strength was modest, these measures provide valuable insight into patients’ functional status and physiological reserve. Incorporating these simple and practical assessments into preoperative evaluation may enhance risk stratification and support targeted interventions to improve recovery and reduce hospital stay length.

## Figures and Tables

**Table 1:** Baseline characteristics among patients admitted for open-heart surgery using Chi-square test and Independent Sample T-test (n = 108).

Variable	Complication		T (Test)/χ^2^	*p*-Value
	Complication (n = 51)	No Complication (n = 57)		
Sex			0.060	0.737
Male (%)	36 (70.59)	39 (68.42)		
Female (%)	15 (29.41)	18 (31.58)		
Type of surgery			7.886	0.019
CABG	25 (49.02)	42 (73.68)		
Valvular replacement	15 (29.41)	11 (19.30)		
Combined	11 (21.57)	4 (7.02)		
**Variable**	**Mean ± S.D.**	**Mean ± S.D.**	**T(test)/**χ^2^	***p*** **-value**
Age (yrs)	66.75 ± 9.90	60.86 ± 10.40	3.004	0.003
BMI (kg/m^2^)	23.48 ± 4.91	24.82 ± 4.43	−1.491	0.139
SPPB (score)	9.41 ± 2.72	10.40 ± 2.24	−2.075	0.040
TUG (s)	13.65 ± 6.75	10.15 ± 4.32	3.239	0.002
5STS (s)	13.37 ± 6.24	10.89 ± 4.24	2.434	0.017
5MWT (m/s^2^)	0.90 ± 0.40	1.07 ± 0.40	−2.283	0.024
Handgrip strength (kg)	24.84 ± 7.89	29.92 ± 7.69	−3.388	0.001

BMI; Body Mass Index, SPPB; short physical performance battery, TUG; timed up and go test, 5STS; Five Times Sit to Stand, 5MWT; 5 m walk test.

**Table 2:** Correlations between physical performance and length of stay in the hospital, and postoperative complications among patients admitted for open-heart surgery, using Spearman’s rank correlation.

	Age *r* (*p*-Value)	SPPB *r* (*p*-Value)	5STS *r* (*p*-Value)	TUG *r* (*p*-Value)	5MWT *r* (*p*-Value)	HG *r* (*p*-Value)
Complications ^a^	0.317 (0.001)	−0.204 (0.034)	0.185 (0.056)	0.285 (0.003)	−0.214 (0.026)	−0.328 (0.001)
Length of hospital stay (n = 103)	0.109 (0.274)	−0.275 (0.005)	0.233 (0.018)	0.253 (0.010)	−0.284 (0.004)	−0.290 (0.003)

^a^ Complication: 0 = no postoperative complication, 1 = postoperative complication. SPPB; short physical performance battery, TUG; timed up and go test, 5STS; Five Times Sit to Stand, 5MWT; 5 m walk test, HG; handgrip strength.

**Table 3:** Results of hierarchical linear regression analysis predicting length of stay in the hospital (n = 103).

Regression Model		B	β	T	*VIF*	R^2^	*F*-Value	Δ R^2^
**Step 1**					0.074	4.020 *	0.074	4.020 *
** Age**	0.028	0.060	0.623					
** Sex ^a^**	2.901	0.269	2.793 **					
**Model**								
** Model 1**					0.134	5.104 *	0.060	6.805 *
** SPPB**	−0.510	−0.251	−2.609 *	1.058				
** Model 2**					0.126	4.739 **	0.051	5.791 *
** 5STS**	0.226	0.246	2.416 *	1.144				
** Model 3**					0.121	4.527 **	0.046	5.203 *
** 5MWT**	−2.711	−0.225	−2.281 *	1.099				
** Model 4**					0.118	4.434 **	0.044	4.943 *
** Handgrip**	−0.163	−0.259	−2.223 *	1.527				
** Model 5**					0.111	4.129 **	0.037	4.096 *
** TUG**	0.172	0.203	2.024 *	1.124				

^a^ sex: male = 1, female = 2 5STS; Five Times Sit to Stand, SPPB; short physical performance battery, TUG: Time up and go, 5MWT; 5 m walk test, VIF; variance inflation factorable drawn. Model 1: Age, Sex + SPPB; Model 2: Age, Sex + 5STS; Model 3: Age, Sex + 5MWT; Model 4: Age, Sex + Handgrip Strength; Model 5: Age, Sex + TUG. * *p*-value < 0.05, ** *p*-value < 0.01.

## Data Availability

The original contributions presented in this study are included in the article. Further inquiries can be directed to the corresponding author.
